# Protective Effects of miR-16-5p and miR-142-3p on Inflammation and Autophagy in Human Corneal Epithelial Cells Under Hyperosmotic Stress In Vitro

**DOI:** 10.3390/ijms27010422

**Published:** 2025-12-31

**Authors:** Min-Ji Cha, Hyunsoo Cho, Yeji Yeon, Yu Jeong Kim

**Affiliations:** 1 Department of Ophthalmology, Hanyang University College of Medicine, Seoul 04763, Republic of Korea; 2 Department of Ophthalmology, Hanyang University Seoul Hospital, Seoul 04763, Republic of Korea; 3 Hanyang Institute of Bioscience and Biotechnology, Hanyang University, Seoul 04763, Republic of Korea

**Keywords:** autophagy, inflammation, miR-16-5p, miR-142-3p, oxidative stress

## Abstract

To investigate the regulatory effects of miR-16-5p and miR-142-3p on inflammation and autophagy in human corneal epithelial cells (HCEpiCs) exposed to hyperosmotic stress, a key pathogenic condition in dry eye disease, HCEpiCs were cultured under NaCl-induced hyperosmotic conditions (450 mOsm, 24 h) and transfected with miR-16-5p or miR-142-3p mimics. Expression of inflammatory cytokines (IL-1β, IL-6, TNF-α, IRAK1), autophagy-related genes (ATG5, Beclin-1, ATG16L1, p62), and apoptotic markers (Bax, Bcl-2, caspase-3) was analyzed by qRT-PCR and Western blot. Reactive oxygen species (ROS), autophagic vesicles, and apoptosis were evaluated using DCFH-DA, DAPRed, and Annexin V assays. The expression levels of antioxidant proteins (SOD1, catalase, NRF2) were also measured. Hyperosmotic stress induces marked inflammatory activation and excessive autophagy in HCEpiCs, accompanied by increased ROS generation and apoptosis. Overexpression of miR-16-5p or miR-142-3p significantly attenuated these effects by suppressing NF-κB-mediated cytokine expression and downregulating ATG5 and ATG16L1 expression, while restoring p62 expression. Both miRNAs reduced oxidative stress and COX-2 expression, enhanced antioxidant defenses, and normalized the expression of apoptotic markers. miR-16-5p and miR-142-3p are important regulators of inflammation and autophagy under hyperosmotic stress. Our findings suggest that modulating intracellular miR-16-5p and miR-142-3p levels in corneal epithelial cells may represent a potential approach to protect the ocular surface under hyperosmotic stress, although their systemic roles in autoimmune dry eye require further clarification.

## 1. Introduction

Sjögren’s syndrome (SS) is a chronic autoimmune exocrinopathy that primarily affects the lacrimal and salivary glands, resulting in severe dry eye and dry mouth symptoms [1,2,3]. The ocular manifestations of SS substantially impair patients’ quality of life and can lead to corneal epithelial damage, visual disturbances, and increased susceptibility to ocular infections [4]. The pathophysiology of SS-associated dry eye involves complex interactions among autoimmune inflammation, secretory gland dysfunction, and tear film instability, ultimately leading to ocular surface hyperosmolarity [5].

Hyperosmotic stress is the central mechanism underlying the pathogenesis of dry eye disease [6,7]. Elevated tear osmolarity induces corneal and conjunctival epithelial injury through multiple interconnected pathways, including the activation of pro-inflammatory cascades, generation of reactive oxygen species (ROS), and initiation of programmed cell death [8]. Recent studies have demonstrated that hyperosmolar stress also disrupts autophagic flux in corneal epithelial cells, thereby exacerbating oxidative stress and apoptosis and contributing to the self-perpetuating cycle of ocular surface damage [3,6,9].

MicroRNAs (miRNAs) are small, non-coding RNAs that post-transcriptionally regulate gene expression and play pivotal roles in inflammation, autophagy, oxidative stress, and cell survival [10,11]. Aberrant miRNA expression has been reported in various ocular surface disorders, including dry eye disease [12]. Our prior tear miRNA profiling study revealed that several immune-associated miRNAs, including miR-16-5p and miR-142-3p, were elevated in SS compared with controls [13].

Functionally, miR-16-5p has been shown to suppress NF-κB-dependent inflammatory responses by targeting IKKβ and IRAK1, and to regulate apoptosis through modulation of BCL-2 [14]. MiR-142-3p modulates immune responses via the TLR4/NF-κB pathway and controls autophagy by targeting ATG16L1 [15,16]. These findings suggest that both miRNAs may participate in the integrated regulatory network of inflammation and autophagy underlying SS-associated dry eye; however, their specific roles in ocular hyperosmolar stress remain largely unknown.

Therefore, we hypothesized that miR-16-5p and miR-142-3p mitigate the deleterious effects of hyperosmotic stress on human corneal epithelial cells (HCEpiCs). In this study, we aimed to elucidate the functional roles of miR-16-5p and miR-142-3p in human corneal epithelial cells exposed to hyperosmotic stress, with a particular focus on their regulatory effects on the inflammatory and autophagic signaling pathways.

## 2. Results

### 2.1. Identification of Candidate Tear miRNAs in Sjögren’s Syndrome

**Figure 1:**
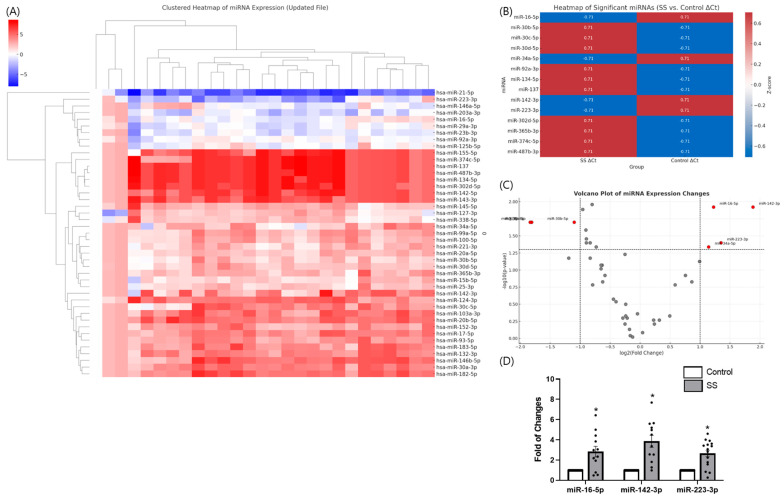
Differential expression of tear microRNAs in Sjögren’s syndrome (SS). (**A**) Hierarchical clustering heatmap of 43 candidate miRNAs in tear samples from patients with SS versus healthy controls, showing distinct separation between groups. (**B**) Heatmap of significantly altered miRNAs between SS and control groups based on ΔCt values. Z-score-normalized expression levels are presented, with red indicating upregulation and blue indicating downregulation in SS tears. (**C**) Volcano plot analysis highlighting significantly dysregulated miRNAs. miR-16-5p, miR-142-3p, and miR-223-3p were identified as top candidates. (**D**) qPCR validation confirmed robust upregulation of miR-16-5p, miR-142-3p, and miR-223-3p in SS tears. Data are expressed as mean ± SEM. Each dot represents an individual biological replicate. * *p* < 0.05.

In our previous study, we performed a custom qPCR array of 43 candidate miRNAs in tear samples from patients with Sjögren’s syndrome (SS) and healthy controls [13]. Hierarchical clustering and heatmap analysis clearly separated patients with SS from controls, indicating distinct miRNA expression signatures (Figure 1A). Differential expression analysis identified 14 miRNAs with significant differences between the two groups (*p* < 0.05) (Figure 1B). Of these, miR-16-5p, miR-142-3p, and miR-223-3p were the most prominent candidates.

Volcano plot analysis revealed that miR-142-3p, miR-16-5p, and miR-223-3p were the most significantly altered (Figure 1C). Independent qPCR validation confirmed a marked upregulation of miR-142-3p (fold change > 4.0, *p* = 0.012), followed by miR-16-5p and miR-223-3p (Figure 1D).

These findings suggest that specific tear miRNAs, particularly miR-16-5p and miR-142-3p, may play key roles in the ocular pathogenesis of SS and provide a rationale for the present in vitro study.

### 2.2. Establishment of a Hyperosmotic Stress Model in Human Corneal Epithelial Cells

**Figure 2:**
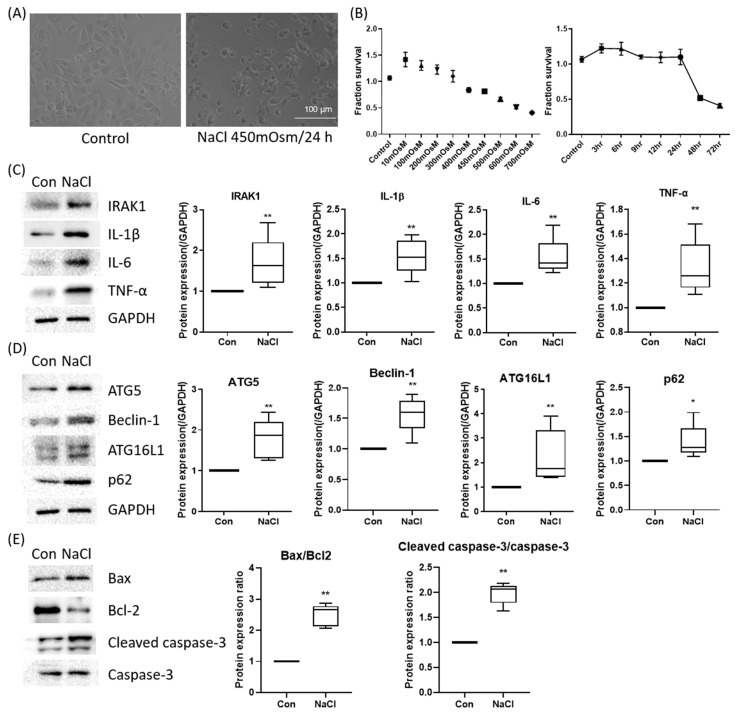
Establishment and molecular validation of a hyperosmolar stress model in human corneal epithelial (hCE) cells. (**A**) Representative phase-contrast morphology of HCEpiCs under iso-osmotic control and hyperosmotic stress (450 mOsm NaCl, 24 h). Scale bar = 100 μm. (**B**) Cell viability analysis (CCK-8) revealed a dose- and time-dependent reduction, with ~30% viability loss at 450 mOsm for 24 h, selected as the optimal condition. (**C**) Western blot analysis demonstrated induction of inflammatory mediators (IL-1β, IL-6, TNF-α, IRAK1). (**D**) Hyperosmotic stress upregulated autophagy-related proteins (ATG5, Beclin-1, ATG16L1) and altered p62 expression, indicating autophagic activation. GAPDH served as the loading control, and all protein levels were normalized to GAPDH. (**E**) Apoptotic signaling was evident with Bax upregulation, Bcl-2 suppression, and increased cleaved caspase-3. * *p* < 0.05, ** *p* < 0.01 versus control.

To recapitulate the hyperosmotic stress associated with dry eye, HCEpiCs were exposed to NaCl-induced hyperosmolar conditions (100–1000 mOsm) for up to 72 h. Treatment with 450 mOsm (NaCl-supplemented medium) for 24 h induced characteristic morphological changes, including cell shrinkage and loss of epithelial integrity (Figure 2A). Cell viability decreased in a dose- and time-dependent manner, with an approximately 30% reduction observed at 450 mOsm for 24 h; therefore, this was selected as the optimal condition for subsequent experiments (Figure 2B).

Western blot analysis demonstrated that hyperosmotic stress (450 mOsm, 24 h) induced robust inflammatory signaling, as evidenced by increased expression of IRAK1, IL-1β, IL-6, and TNF-α (Figure 2C). The autophagy-related proteins ATG5, Beclin-1, ATG16L1, and p62 were also upregulated, which was consistent with activation of the autophagic machinery (Figure 2D). Furthermore, Bax expression increased, whereas that of the anti-apoptotic protein Bcl-2 decreased, indicating the activation of apoptotic pathways (Figure 2E). Collectively, these results confirm that hyperosmotic stress triggers a multifaceted stress response involving inflammation, autophagy, and apoptosis in corneal epithelial cells.

### 2.3. miR-16-5p and miR-142-3p Suppress Hyperosmolarity-Induced Inflammation

**Figure 3:**
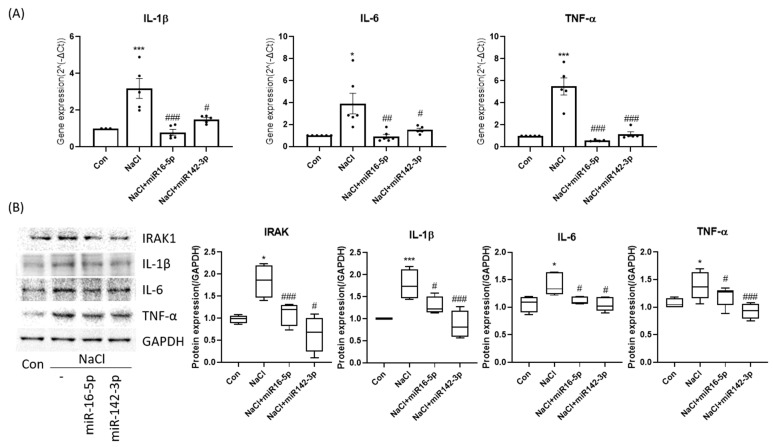
miR-16-5p and miR-142-3p repress hyperosmolarity-induced inflammatory signaling. (**A**) qPCR analysis of IL-1β, IL-6, and TNF-α expression in HCEpiCs under hyperosmotic stress with or without miR-16-5p/miR-142-3p mimic transfection. Data are presented as mean ± SEM. Each dot represents an individual biological replicate. (**B**) Western blot analysis and densitometric quantification showing that both miRNAs significantly attenuated cytokine induction (IL-1β, IL-6, TNF-α, IRAK1). GAPDH served as the loading control, and all protein levels were normalized to GAPDH. * *p* < 0.05, *** *p* < 0.001 versus control. # *p* < 0.05, ## *p* < 0.01, ### *p* < 0.001 versus NaCl group.

To assess the functional roles of selected miRNAs, HCEpiCs were transfected with miR-16-5p or miR-142-3p mimics under hyperosmotic stress. qPCR and Western blot analyses revealed that both miRNAs significantly attenuated the induction of IL-1β, IL-6, TNF-α, and IRAK1 (Figure 3A,B). These results indicate that miR-16-5p and miR-142-3p negatively regulate hyperosmolarity-induced pro-inflammatory signaling.

### 2.4. Regulation of Autophagy by miR-16-5p and miR-142-3p

**Figure 4:**
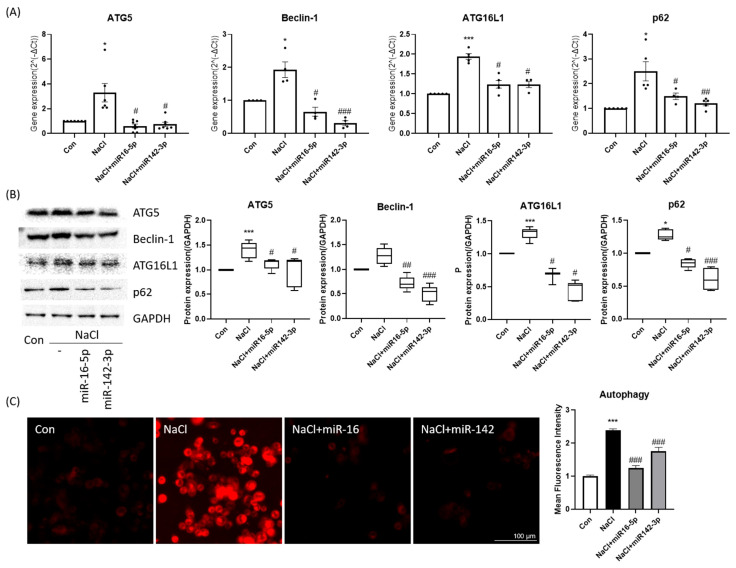
Autophagy regulation by miR-16-5p and miR-142-3p in HCEpiCs. (**A**) qPCR analysis of autophagy-related genes (ATG5, Beclin-1, ATG16L1, p62) in hyperosmotic stress ± miRNA mimics. Data are presented as mean ± SEM. Each dot represents an individual biological replicate. (**B**) Western blot analysis and quantification of autophagy proteins (ATG5, Beclin-1, ATG16L1, p62). GAPDH served as the loading control, and all protein levels were normalized to GAPDH. (**C**) Representative DAPRed fluorescence imaging of autophagic vesicles (red) and quantitative analysis of fluorescence intensity. Both miRNAs reduced autophagy marker expression and vesicle accumulation under hyperosmotic conditions. * *p* < 0.05, *** *p* < 0.001 versus control. # *p* < 0.05, ## *p* < 0.01, ### *p* < 0.001 versus NaCl group.

Analysis of autophagy markers showed that hyperosmotic stress increased ATG5, Beclin-1, and ATG16L1 expression while altering p62 levels. Overexpression of miR-16-5p or miR-142-3p markedly reduced the mRNA and protein levels of these autophagy-related genes and partially restored p62 expression (Figure 4A,B). DAPRed fluorescence imaging revealed that hyperosmotic stress markedly increased autophagic vesicle formation, which was substantially reduced following treatment with either miRNA mimic alone (Figure 4C). These results suggest that both miRNAs counteract excessive autophagy induced by hyperosmotic stress.

### 2.5. miR-16-5p and miR-142-3p Reduce Oxidative Stress and Apoptosis

**Figure 5:**
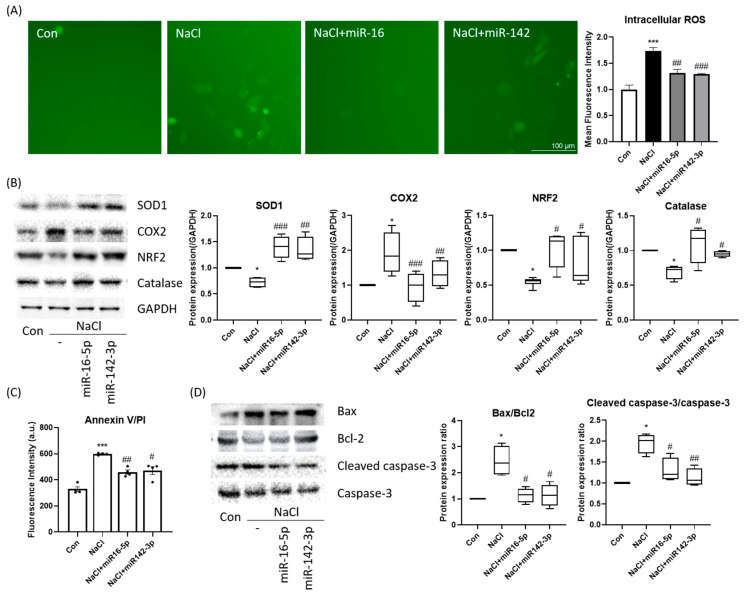
miR-16-5p and miR-142-3p suppress oxidative stress and apoptosis. (**A**) ROS detection using DCFH-DA (green) demonstrated increased oxidative stress in hyperosmotic cells, which was attenuated by miRNA mimic treatment. Quantitative fluorescence intensity is shown. (**B**) Western blot analysis of antioxidant and stress-related proteins (SOD, COX2, NRF2, catalase) GAPDH served as the loading control, and all protein levels were normalized to GAPDH. (**C**) Annexin V-FITC/PI apoptosis assay showing reduced apoptotic cell population following mimic treatment. All data are presented as mean ± SEM. Each dot represents an individual biological replicate. (**D**) Western blot analysis of apoptotic markers (Bax, Bcl-2, cleaved caspase-3, caspase-3) and corresponding quantification of Bax/Bcl-2 and cleaved caspase-3/caspase-3 ratios. All data are presented as mean ± SEM. *** *p* < 0.001, * *p* < 0.05 versus control. ### *p* < 0.001, ## *p* < 0.01, # *p* < 0.05 versus NaCl group.

Intracellular ROS levels, as assessed by DCFH-DA fluorescence, were significantly elevated under hyperosmotic stress but were markedly reduced upon mimic transfection (Figure 5A). Western blot analysis confirmed increased antioxidant protein expression (SOD1, catalase, NRF2) in the mimic-treated groups, along with reduced COX-2 induction (Figure 5B). Furthermore, apoptosis assays demonstrated that mimic treatment lowered Annexin V-positive cell populations compared with NaCl-only treatment (Figure 5C). At the molecular level, the mimics decreased Bax and cleaved caspase-3 while restoring Bcl-2 expression, resulting in reduced Bax/Bcl-2 and cleaved caspase-3/caspase-3 ratios (Figure 5D). These findings indicate that miR-16-5p and miR-142-3p suppress oxidative stress and apoptotic signaling, thereby promoting epithelial cell survival.

## 3. Discussion

In this study, hyperosmotic stress (450 mOsm) triggered inflammation, autophagy, oxidative stress, and apoptosis in HCEpiCs, while overexpression of miR-16-5p or miR-142-3p significantly suppressed these responses by inhibiting NF-κB-mediated inflammation, reducing oxidative and autophagic activity, and preventing cell death. Our bioinformatic analysis predicted enrichment of NF-κB, autophagy, and oxidative stress pathways, consistent with the observed in vitro phenotypes. These predicted target pathways align with the molecular changes observed under hyperosmotic stress and support the functional relevance of miR-16-5p and miR-142-3p in regulating these stress responses.

Overexpression of either miR-16-5p or miR-142-3p significantly reduces cytokine expression, indicating suppression of the inflammatory cascade [17,18]. These results are consistent with the known roles of these miRNAs in the regulation of inflammatory signaling. In systemic autoimmune conditions such as primary Sjögren’s syndrome, miR-16-5p has been described as part of broader immune-activation signatures rather than as a direct regulator of disease-specific autoantigen pathways. Its systemic autoimmune associations are therefore distinct from the acute epithelial hyperosmotic stress responses examined in our model, and our study does not address Ro52/TRIM21-related mechanisms. For example, miR-16 can directly target key upstream mediators of the NF-κB pathway (such as the IKKα/β kinase complex), thereby blocking the cascade that leads to inflammatory gene transcription [19]. Consistently, He et al. found that miR-16-5p overexpression in corneal epithelial cells led to reduced cytokine production by inhibiting NF-κB activation (e.g., via TLR4/IKK pathways) [20,21]. In contrast, miR-142-3p has been reported to play context-dependent roles. While some immune-cell studies suggest a pathogenic function in Sjögren’s syndrome, epithelial studies show anti-inflammatory effects via targeting IRAK1 [22]. Thus, although miR-16-5p and miR-142-3p act through distinct upstream nodes, both converge on attenuating NF-κB-mediated inflammatory signaling in corneal epithelial cells under acute hyperosmotic stress.

Hyperosmolar stress is known to elevate intracellular ROS levels, leading to oxidative damage to lipids, proteins, and DNA [23,24]. Excess ROS not only harms cells directly but also further activates stress pathways like NF-κB, creating a vicious cycle that can trigger cell death [25,26,27]. In our model, the miR-16-5p and miR-142-3p mimics significantly lowered ROS levels. The miRNA treatments also reduced COX-2 expression, an inducible pro-inflammatory enzyme that is typically upregulated by NF-κB in response to stress [28]. COX-2 contributes to inflammatory and oxidative injury on the ocular surface; therefore, its suppression by miRNAs is a beneficial outcome, likely stemming from NF-κB pathway inhibition [26,29]. By blocking NF-κB activation, miR-16-5p and miR-142-3p break the feed-forward loop between inflammation and oxidative stress, thereby reducing downstream COX-2 and pro-oxidant factors. Additionally, we noted increased levels of protective antioxidant proteins, such as SOD1, catalase, and the transcription factor NRF2 (a master regulator of antioxidant responses), following miR-16-5p or miR-142-3p mimic treatment. This suggests that these miRNAs help tilt the balance back toward a protective antioxidant state. The enhanced NRF2 and antioxidant enzyme expression is consistent with prior findings that miR-16-5p can activate antioxidative pathways; for example, our previous study reported that miR-16-5p attenuates oxidative stress in ocular cells in part by upregulating the NRF2 pathway [30].

Autophagy generally serves as a cytoprotective mechanism that removes damaged organelles and misfolded proteins, and previous studies have shown that its activation can protect corneal epithelial cells from inflammatory injury under desiccation or hyperosmolar stress [7,31,32,33]. However, in our model, hyperosmotic stress appeared to induce excessive or dysregulated autophagy, which may have contributed to epithelial damage rather than protection. Consistent with previous findings, many studies report that autophagy-related miRNAs modulate this pathway via NF-κB or Beclin-1 regulation [7]. The observed effects of miR-16-5p and miR-142-3p suggest that these miRNAs mitigate the hyperosmolarity-induced disturbances in autophagic processes. Our findings and prior evidence indicate that miR-16-5p regulates autophagy indirectly through mTOR/NF-κB-associated pathways [34], whereas miR-142-3p directly targets ATG5 and ATG16L1 [35], consistent with its ability to reduce their expression and limit excess autophagic activity.

The role of autophagy in dry eye is inherently complex; it can be protective when appropriately activated, yet detrimental when excessive or defective, as seen in disease-associated ATG16L1 polymorphisms [20,36,37]. Previous studies have suggested that inhibition of specific miRNAs can restore autophagic flux and reduce oxidative stress in experimental models of dry eye [13,16]. However, in our study, the upregulation of miR-142-3p was beneficial, indicating a possible context-dependent effect. This discrepancy likely reflects differences in the experimental context and timing; in chronic in vivo conditions, miR-142-3p may be pathologically overexpressed, whereas in our acute in vitro model, its transient elevation may have compensated for early stress-induced depletion. Both studies underscore that miR-142-3p is a key regulator of autophagy and oxidative stress on the ocular surface [38]. Further studies are needed to investigate the time-dependent and context-specific roles of miR-142-3p to clarify its dual function in ocular surface homeostasis. Several tear miRNA profiling studies in primary Sjögren’s syndrome have consistently reported upregulation of immune-associated miRNAs, including miR-16-5p and miR-142-3p, distinguishing autoimmune dry eye from non-autoimmune forms [13,39]. Our prior tear miRNA study similarly demonstrated increased expression of these miRNAs in patients with Sjögren’s syndrome compared with healthy controls, supporting their relevance as extracellular biomarkers in the autoimmune context. Moreover, NOD-based Sjögren’s models exhibit altered tear or lacrimal gland miRNA signatures involving miR-142-3p, correlating with immune infiltration and glandular dysfunction [40]. These findings position miR-16-5p and miR-142-3p within established autoimmune dry eye signatures [41] while underscoring that their intracellular roles in acute epithelial stress—examined in our study—represent a distinct biological context. To bridge these model-specific differences, we plan to evaluate tear and tissue miRNA alterations in a Sjögren-like NOD mouse model in future studies.

Our in vitro findings suggest that hyperosmotic stress leads to a decrease in intracellular miR-16-5p and miR-142-3p, relieving suppression of IRAK1/NF-κB and ATG16L1 pathways and consequently enhancing inflammation, oxidative stress, autophagy, and epithelial cell death. In contrast, tear samples from patients with SS showed elevated extracellular levels of these miRNAs, which appear to be inconsistent with the in vitro results. This discrepancy likely reflects compartment- and context-dependent miRNA regulation. Acute hyperosmotic stress may reduce intracellular miRNA levels while promoting their export, whereas chronic autoimmune inflammation in SS leads to sustained extracellular accumulation. Thus, elevated tear miRNAs in SS represent chronic inflammatory signatures rather than evidence supporting therapeutic upregulation. Stress-induced selective export of miRNAs via extracellular vesicles or protein complexes may deplete intracellular pools, while increasing their release into tears, either as a protective paracrine signal or as a consequence of cellular damage [13,42]. Moreover, miRNA dynamics may differ over time. Acute stress may cause an early intracellular decline that triggers inflammatory and oxidative cascades, whereas chronic stress or cellular turnover in long-standing SS may enhance miRNA biogenesis and extracellular accumulation. Finally, tear miRNAs originate from multiple ocular and glandular sources beyond the corneal epithelium, including conjunctival cells, lacrimal glands, and infiltrating immune cells, which could collectively elevate tear miRNA levels even when corneal epithelial expression is reduced [13,43].

It is important to emphasize that our in vitro data pertain specifically to intracellular miRNA levels in corneal epithelial cells under acute hyperosmotic stress, whereas previous studies in Sjögren’s syndrome have predominantly described systemic or glandular overexpression of miR-142-3p and miR-16-5p in T cells, salivary gland tissue, and peripheral blood. In those settings, miR-142-3p acts as a T-cell–derived exosomal effector that impairs cAMP/Ca^2+^ signaling and exocrine secretion, and targeted inhibition (e.g., antagomirs, exosome-based antagonists) has been proposed as a therapeutic strategy. By contrast, in our acute epithelial model hyperosmotic stress appears to be associated with a relative depletion of intracellular miR-16-5p and miR-142-3p in corneal epithelial cells, and transient mimic transfection counteracts NF-κB-driven inflammation, oxidative stress, and dysregulated autophagy. Thus, our data should be interpreted as evidence for cell-intrinsic, context-dependent regulatory functions, rather than a direct recommendation to augment miR-142-3p systemically in Sjögren’s syndrome.

Several studies investigating tear miRNA profiles in patients with Sjögren’s syndrome have consistently demonstrated that immune-associated miRNAs—including miR-16-5p and miR-142-3p—are selectively elevated compared with non-autoimmune dry eye, forming a characteristic autoimmune tear signature linked to immune activation and glandular dysfunction [13,40]. Similar alterations have been reported in Sjögren’s-like murine models, including NOD-derived models, in which changes in tear or lacrimal gland miRNAs correlate with lymphocytic infiltration and declining secretory function [41]. These findings position miR-16-5p and miR-142-3p within established Sjögren’s-related regulatory networks and indicate that their extracellular tear profiles reflect chronic immune-mediated processes, which are distinct from the acute, intracellular stress responses observed in our hyperosmotic epithelial model [42]. Integrating these data provides important context for interpreting the compartment-specific and condition-dependent roles of these miRNAs in autoimmune versus non-autoimmune dry eye.

Taken together, these observations underscore the need for careful interpretation of miRNA dynamics across different biological compartments and disease contexts, which also informs the limitations of our current model.

This study has some limitations. First, it was conducted using an in vitro hyperosmotic stress model with human corneal epithelial cells, which cannot fully replicate the complex in vivo microenvironment of Sjögren’s syndrome-associated dry eye, including immune cell infiltration, glandular dysfunction, and tear film instability. Second, direct validation of the target interactions (e.g., IRAK1 and ATG16L1) was not performed. In addition, choline chloride-matched osmotic controls were not included, representing a methodological limitation. Incorporating ion-substitution osmotic controls in future studies will help distinguish Na^+^-specific effects from hyperosmolarity-driven responses. Third, this study primarily examined intracellular miRNA changes, whereas clinical samples measured tear or extracellular miRNA levels, introducing a compartmental difference that may have contributed to the discrepancies between the in vitro and in vivo data. Finally, this study employed a single cell line and a short-term exposure model. Thus, long-term or in vivo investigations are required to confirm whether modulating these miRNAs can restore ocular surface homeostasis under physiological conditions.

Furthermore, because miR-16-5p has been implicated in autoantibody-associated immune activation and has been linked to Ro52/TRIM21 in other autoimmune conditions, it will be important in future work to determine whether tear or ocular surface miR-16-5p correlates with Ro/La autoantibody titers and Ro52/TRIM21 expression in patients with Sjögren’s syndrome. These analyses were beyond the scope of the present in vitro study but would provide important mechanistic context regarding the immunologic relevance of miR-16-5p dysregulation.

Despite these limitations, our findings suggest that intracellular modulation of miR-16-5p and miR-142-3p may help attenuate epithelial stress responses under hyperosmotic conditions, although their therapeutic relevance in autoimmune dry eye requires further in vivo investigation.

## 4. Materials and Methods

### 4.1. Cell Lines and Cell Culture

Human corneal epithelial cells (HCEpiCs) were purchased from Innoprot (Derio, Bizkaia, Spain) and maintained in Corneal Epithelial Cell Medium (Innoprot, Derio, Bizkaia, Spain) supplemented with 5% fetal bovine serum (FBS), 100 U/mL penicillin, and 100 μg/mL streptomycin. The cells were cultured at 37 °C in a humidified incubator with 5% CO_2_.

### 4.2. In Vitro Hyperosmotic Stress Model

To mimic dry eye-like conditions in vitro, hyperosmotic stress was induced in HCEpiCs by adding sodium chloride (NaCl) to the culture medium. After a stable subculture, the cells were seeded into 6-well plates and switched to serum-free medium (DMEM/F12 without FBS) for 24 h. The osmolality was adjusted to 450 ± 5 mOsm by titrating a 5 M NaCl stock solution into serum-free medium, and the final osmolality was confirmed using a freezing-point osmometer [44,45,46].

### 4.3. MicroRNA Transfection

Transfection of miRNA mimics was performed using INTERFERin® reagent (Polyplus, Illkirch, France) according to the manufacturer’s protocol. Briefly, cells were seeded at a density of 3 × 10^5^ cells per 60 mm culture plate. The reagent was diluted in serum-free medium and mixed with the designated miRNA mimics. The complexes were added to cells in fresh medium, and after 4 h of incubation at 37 °C in 5% CO_2_, the medium was replaced with conditioned fresh medium.

- hsa-miR-16-5p mimic: UAGCAGCACGUAAAUAUUGGCG
- hsa-miR-142-3p mimic: UGUAGUGUUUCCUACUUUAUGGA

### 4.4. RNA Extraction, cDNA Synthesis, and Quantitative Real-Time PCR (qRT-PCR)

Total RNA was extracted using the miRNeasy Micro Kit (Qiagen, Hilden, Germany) and reverse-transcribed using the QuantiTect^®^ Reverse Transcription Kit (Qiagen). Quantitative PCR was performed with GreenStar™ qPCR Master Mix on a LightCycler 480 platform (Roche, Basel, Switzerland). The following primer sequences for the target genes (Table 1) were used:

Thermal cycling conditions were: 95 °C for 20 s, followed by 40 cycles of 95 °C for 3 s and 60 °C for 30 s, and a final step at 95 °C for 15 s, 60 °C for 1 min, and 95 °C for 15 s. Threshold cycle (Ct) values were normalized to GAPDH (ΔCt), and relative expression levels were calculated using the 2^–ΔΔCt^ method. All samples were analyzed in triplicate, and the experiments were repeated at least three times.

### 4.5. Western Blot Analysis

The cells were lysed in radioimmunoprecipitation assay (RIPA) buffer (50 mM Tris-HCl, pH 7.4; 150 mM NaCl; 1% NP-40; 0.5% sodium deoxycholate; 0.1% SDS) containing protease and phosphatase inhibitors (Roche, #PPC1010). Lysates were incubated on ice for 20 min and centrifuged at 10,000× *g* for 10 min at 4 °C. Protein concentrations were determined using bicinchoninic acid (BCA) assay (Thermo Fisher Scientific, Waltham, MA, USA). Equal amounts of protein were separated by sodium dodecyl sulfate–polyacrylamide gel electrophoresis (SDS-PAGE) on 12% polyacrylamide gels and transferred to methanol-activated polyvinylidene difluoride (PVDF) membranes (Millipore, Burlington, MA, USA). Membranes were blocked with 5% bovine serum albumin (BSA) in TBS-T (Tris-buffered saline, 0.1% Tween-20) and incubated with primary antibodies (ATG16L1, ATG5, IRAK1, IL-1β, Bax, Bcl-2, IL-6, Beclin-1, TNF-α, COX-2, SOD1, SQSTM1/p62, cleaved caspase-3, caspase-3, and GAPDH; all from Cell Signaling Technology (Danvers, MA, USA) or ABclonal (Woburn, MA, USA). After incubation with horseradish peroxidase (HRP)-conjugated secondary antibodies, bands were detected using enhanced chemiluminescence (ECL, GE Healthcare, Chicago, IL, USA). Band intensities were quantified using the ImageJ software (version 1.53, National Institutes of Health, Bethesda, MD, USA). For all proteins analyzed, densitometric quantification was performed relative to GAPDH, which served as the uniform loading control throughout the study. For proteins with low expression levels, higher protein loading and optimized exposure settings were applied to improve detection sensitivity and band quality, as reflected in the representative blots shown.

### 4.6. Measurement of Intracellular Reactive Oxygen Species (ROS)

Intracellular reactive oxygen species (ROS) levels were determined using a DCFH-DA assay kit (Dojindo, Kumamoto, Japan). HCEpiCs were seeded onto culture plates and incubated overnight. After washing with HBSS, cells were incubated with DCFH-DA working solution (1:1000 dilution) for 30 min at 37 °C. The cells were then exposed to a hyperosmotic medium (450 mOsm NaCl). Fluorescence was observed under a fluorescence microscope (excitation/emission [Ex/Em]: 488/500–550 nm) and quantified using a plate reader (Ex/Em: 490–520/510–540 nm).

### 4.7. Detection of Autophagy

Autophagic activity was measured using a DAPRed Autophagy Detection Kit (Dojindo, Kumamoto, Japan). A 0.1 mmol/L stock solution was prepared in DMSO and diluted to a 0.1 μmol/L working solution with culture medium. The cells were incubated with DAPRed for 30 min at 37 °C, followed by treatment with a hyperosmotic medium (450 mOsm NaCl). Autophagic structures were visualized using a fluorescence microscope (Zeiss, Oberkochen, Germany; Ex: 500–560 nm, Em: 690–750 nm).

### 4.8. Apoptosis Assay

Apoptosis was assessed using the Annexin V Apoptosis Plate Assay Kit (Dojindo, Kumamoto, Japan). Annexin V-FITC was prepared according to the manufacturer’s instructions. The cells were seeded in 96-well black plates and cultured overnight. After exposure to the experimental conditions, the cells were incubated with Annexin V working solution for 15 min at room temperature in the dark. Fluorescence intensity was measured using a microplate reader (BioTek, Winooski, VT, USA; Ex/Em: 488/525 nm).

### 4.9. Cell Viability Assay

Cell viability was measured using the Cell Counting Kit-8 (CCK-8; Dojindo, Kumamoto, Japan). HCEpiCs were seeded into 96-well plates and cultured overnight. Following hyperosmotic treatment (450 mOsm NaCl), 10 μL of CCK-8 reagent was added to each well and incubated for 1–4 h. Absorbance was measured at 450 nm using a microplate reader(BioTek, Winooski, VT, USA). Relative cell viability was normalized to that of untreated controls.

### 4.10. Bioinformatics Analysis of miRNA

Expression profiles of tear microRNAs were analyzed to identify differentially expressed miRNAs in patients with Sjögren’s syndrome (SS) compared with healthy controls. Hierarchical clustering analysis of 43 candidate miRNAs was performed using the pheatmap package in R (version 4.2.1) to visualize overall expression patterns and group segregation between SS and control samples. Heatmap of significantly altered miRNAs were generated using ΔCt values normalized as Z-scores. For this study, miRNA target prediction was performed using TargetScan (https://www.targetscan.org; accessed on 15 November 2024) and miRWalk (http://mirwalk.umm.uni-heidelberg.de; accessed on 11 October 2024), and overlapping predicted targets were selected for downstream path-way analysis. Gene Ontology (GO) and KEGG pathway enrichment analyses were conducted using the clusterProfiler package (version 4.6.2) in R, with significantly enriched pathways defined as those with adjusted *p*-values < 0.05. Ingenuity Pathway Analysis (IPA) was not used in this study.

### 4.11. Experimental Design and Batch Consistency

All experiments were performed using at least three independent biological replicates. For each biological replicate, human corneal epithelial cells derived from the same culture batch and passage were used consistently across miRNA expression analysis, Western blot, ROS measurement, autophagy assays, and apoptosis assays to ensure internal experimental consistency.

When different experimental batches were unavoidably used for specific assays, key molecular outcomes—including the expression patterns of representative inflammatory and autophagy-related proteins—were cross-validated and confirmed to show comparable trends.

For Western blot analyses, selected experiments were repeated with optimized protein loading and exposure conditions to improve band clarity and signal-to-noise ratio. Representative higher-quality blots were included in the main figures, and additional uncropped or alternative blots are provided in the Supplementary Materials.

### 4.12. Statistical Analysis

All experiments were performed with at least three independent biological replicates. Data are expressed as mean ± standard error of the mean (SEM). Statistical analysis was performed using the Wilcoxon signed-rank test for paired data, the Mann–Whitney U test for group comparisons, and the Kruskal–Wallis test for comparisons among multiple groups. Statistical significance was set at *p* < 0.05. All statistical analyses and graphical visualizations were performed using GraphPad Prism (version 10.0; GraphPad Software, San Diego, CA, USA).

## 5. Conclusions

Our findings identify miR-16-5p and miR-142-3p as modulators of inflammation, autophagy, and oxidative stress in human corneal epithelial cell cultures under hyperosmotic conditions in vitro. Future in vivo studies are needed to determine whether selective, compartment-specific modulation of these miRNAs can be safely exploited for the treatment of Sjögren’s syndrome-associated dry eye.

## Figures and Tables

**Table 1:** qRT-PCR primer sequences used in this study.

	Forward (5′→3′)	Reverse (5′→3′)
*IL-1* *β*	CCACAGACCTTCCAGGAGAATG	GTGCAGTTCAGTGATCGTACAGG
*IL-6*	AGACAGCCACTCACCTCTTCAG	TTCTGCCAGTGCCTCTTTGCTG
*TNF-* *α*	CTCTTCTGCCTGCTGCACTTTG	ATGGGCTACAGGCTTGTCACTC
*ATG5*	AGACCTTCTGCACTGTCCATC	GCAATCCCATCCAGAGTTGCT
*BECN1*	AACCAGATGCGTTATGCCCA	TCCATTCCACGGGAACACTG
*LC3B*	AAGGCTTTCAGAGAGACCCTG	CCGTTACCCTGCGTTTGT
*SQSTM1*	TGTGTAGCGTCTGCGAGGGAAA	AGTGTCCGTGTTTCACCTTCCG
*ATG16L1*	CTACGGAAGAGAACCAGGAGCT	CTGGTAGAGGTTCCTTTGCTGC
*GAPDH*	GTCTCCTCTGACTTCAACAGCG	ACCACCCTGTTGCTGTAGCCAA

## Data Availability

The data presented in this study are available on request from the corresponding author. The data have been previously presented in part at a scientific meeting and are not publicly available due to ethical and privacy restrictions.

## Supplementary Materials

The supporting information can be downloaded at: https://www.mdpi.com/article/10.3390/ijms27010422/s1.

## Abbreviations

The following abbreviations are used in this manuscript:

SS	Sjögren’s syndrome
HCEpiC	Human corneal epithelial cell
miRNA	microRNA
ROS	Reactive oxygen species
IL	Interleukin
TNF	Tumor Necrosis factor
ATG	Autophagy-related gene
