# Supplementary material for: Protective Effects of miR-16-5p and miR-142-3p on Inflammation and Autophagy in Human Corneal Epithelial Cells Under Hyperosmotic Stress In Vitro
Source: Int J Mol Sci. 2025 Dec 31;27(1):422. doi: 10.3390/ijms27010422 (PMC12785823; doi:10.3390/ijms27010422)
Supplement: Supplementary file 1 [file ijms-27-00422-s001.zip › ijms-4006903-supplementary.pptx]

## Slide 1
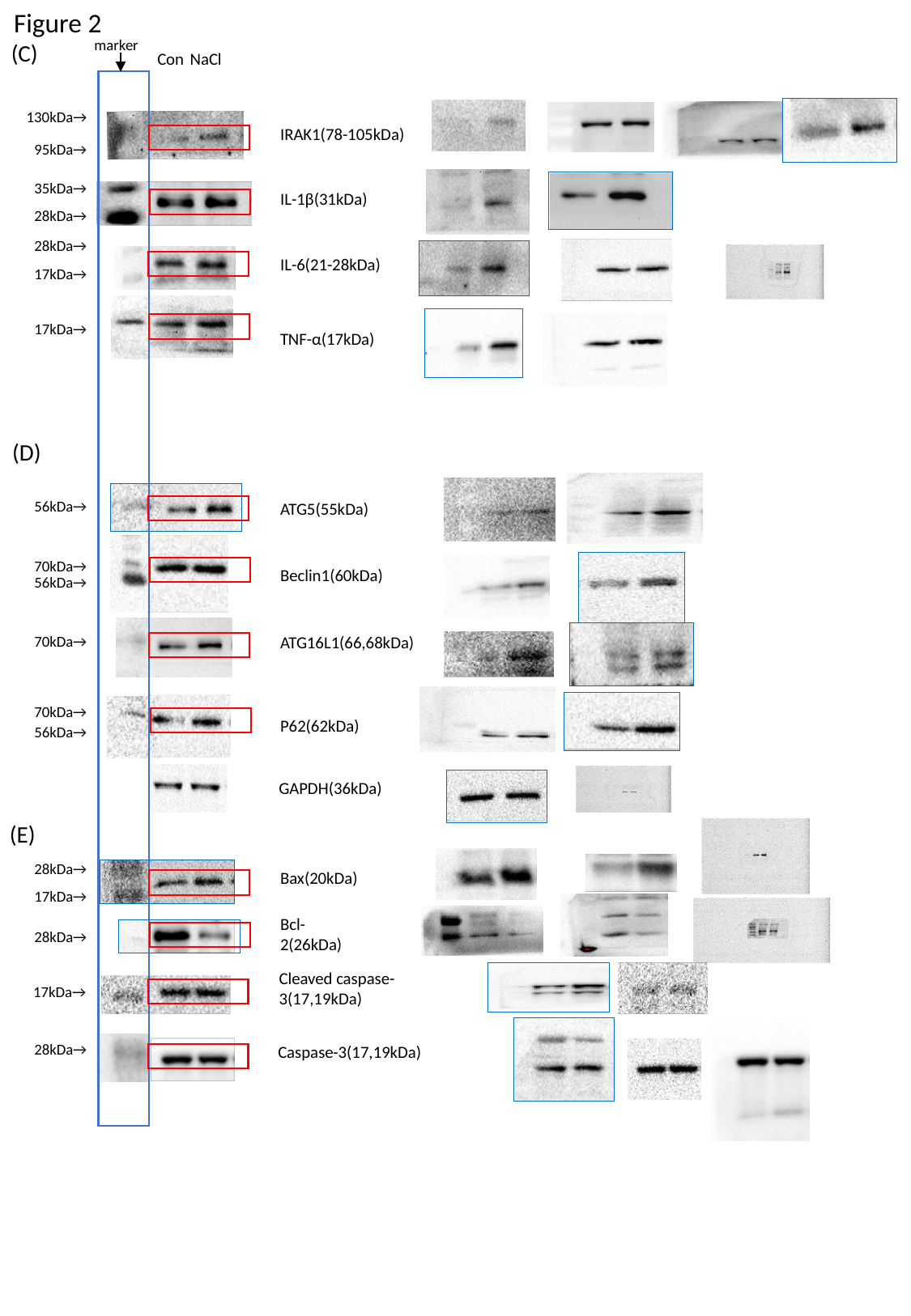

Figure 2
marker
(C)
Con
NaCl
130kDa→
IRAK1(78-105kDa)
95kDa→
35kDa→
IL-1β(31kDa)
28kDa→
28kDa→
IL-6(21-28kDa)
17kDa→
17kDa→
TNF-α(17kDa)
(D)
56kDa→
ATG5(55kDa)
70kDa→
Beclin1(60kDa)
56kDa→
70kDa→
ATG16L1(66,68kDa)
70kDa→
P62(62kDa)
56kDa→
GAPDH(36kDa)
(E)
28kDa→
Bax(20kDa)
17kDa→
Bcl-2(26kDa)
28kDa→
Cleaved caspase-3(17,19kDa)
17kDa→
28kDa→
Caspase-3(17,19kDa)

## Slide 2
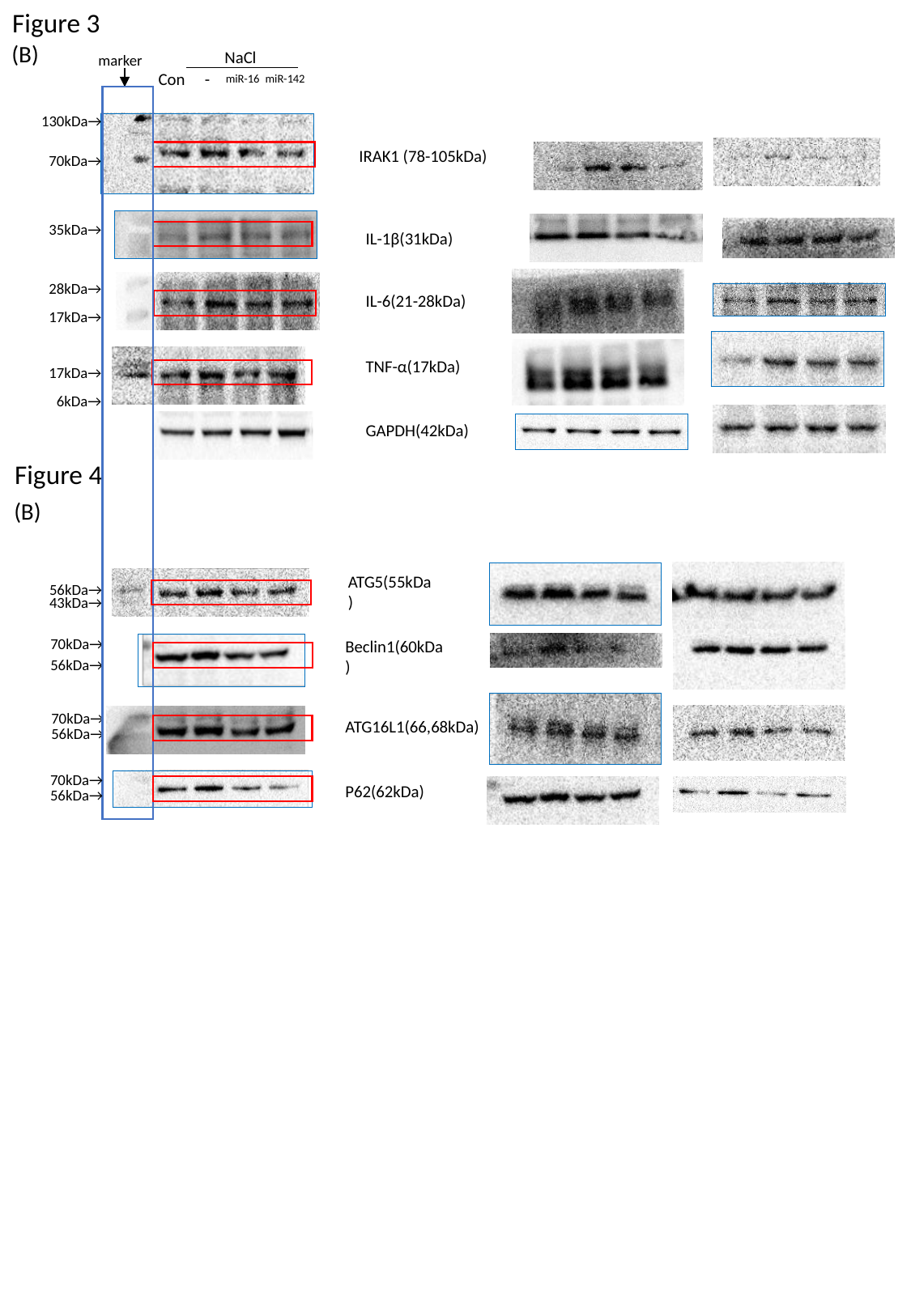

Figure 3
(B)
NaCl
marker
Con
-
miR-16
miR-142
130kDa→
IRAK1 (78-105kDa)
70kDa→
35kDa→
IL-1β(31kDa)
28kDa→
IL-6(21-28kDa)
17kDa→
TNF-α(17kDa)
17kDa→
6kDa→
GAPDH(42kDa)
Figure 4
(B)
56kDa→
ATG5(55kDa)
43kDa→
70kDa→
Beclin1(60kDa)
56kDa→
70kDa→
ATG16L1(66,68kDa)
56kDa→
70kDa→
P62(62kDa)
56kDa→

## Slide 3
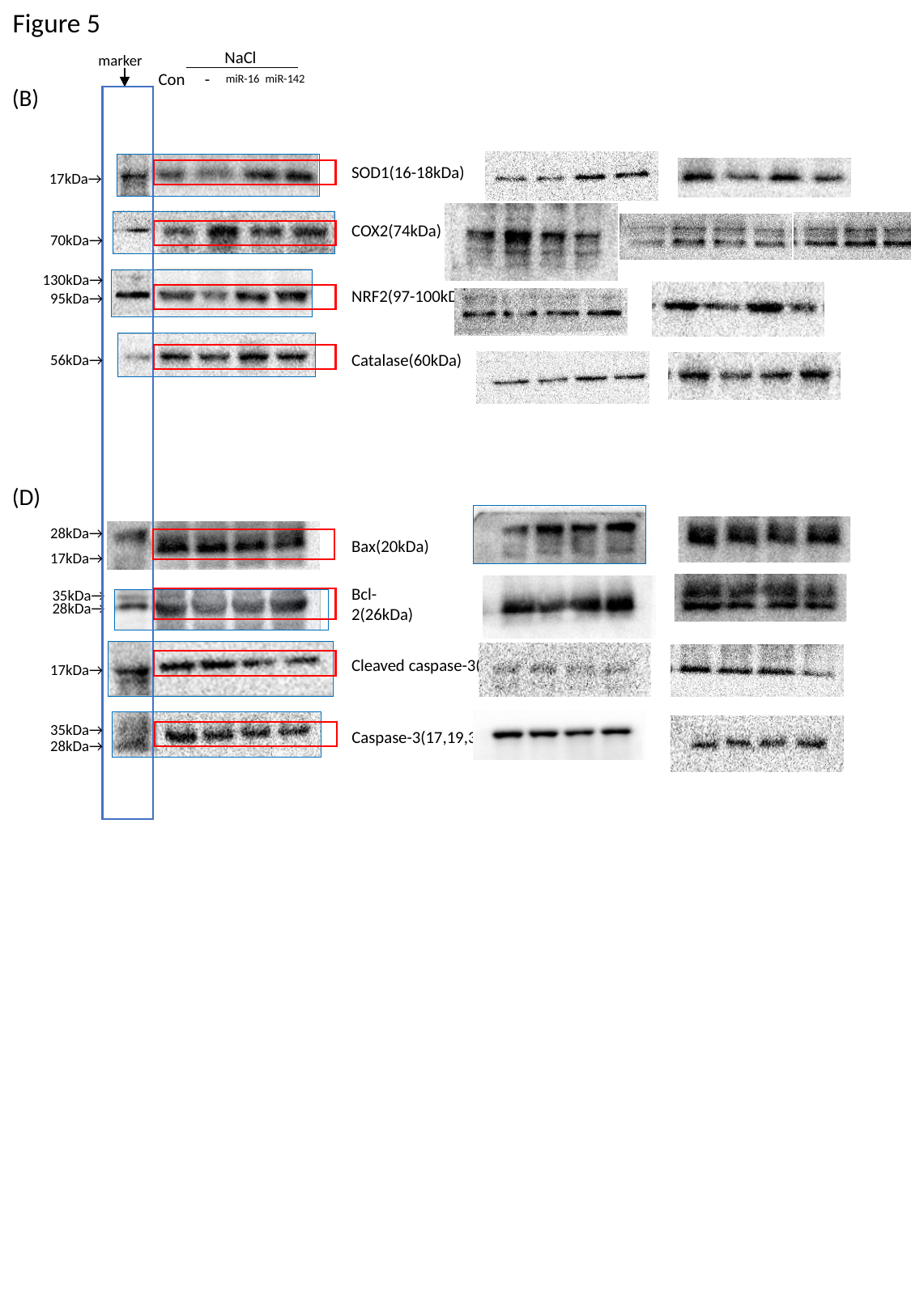

Figure 5
NaCl
marker
Con
-
miR-16
miR-142
(B)
SOD1(16-18kDa)
17kDa→
COX2(74kDa)
70kDa→
130kDa→
NRF2(97-100kDa)
95kDa→
Catalase(60kDa)
56kDa→
(D)
28kDa→
Bax(20kDa)
17kDa→
35kDa→
Bcl-2(26kDa)
28kDa→
Cleaved caspase-3(17,19kDa)
17kDa→
35kDa→
Caspase-3(17,19,35kDa)
28kDa→
